# Temporomandibular joint arthrocentesis. Review of the literature

**DOI:** 10.4317/medoral.17670

**Published:** 2012-02-09

**Authors:** Florencio Monje-Gil, Dorrit Nitzan, Raul González-Garcia

**Affiliations:** 1 Head of the Department of Oral and Maxillofacial Surgery. University Hospital Infanta Cristina. Private practice. Spain; 2 Department of Oral and Maxillofacial Surgery Hadassah School of Dental Medicine. The Hebrew University Jerusalem. Israel; 3 Staff Surgeon of Department of Oral and Maxillofacial Surgery. University Hospital Infanta Cristina. Badajoz. Spain

## Abstract

The treatment of the temporomandibular joint (TMJ) is still controversial. TMJ arthrocentesis represents a form of minimally invasive surgical treatment in patients suffering from internal derangement of the TMJ, especially closed lock. It consists of washing the joint with the possibility of depositing a drug or other therapeutic substance. Resolution of symptoms is due to the removal of chemical inflammatory mediators and changes in intra-articular pressure. Numerous clinical studies regarding this technique have been published. The goal of this paper is to review all clinical articles that have been published with regard to the critique of this technique. 19 articles with different designs fulfilling selection guidelines were chosen. A series of clinical and procedure variables were analyzed. Although the mean of improvement was higher that 80%, further research is needed to determine more homogeneous indications for TMJ athrocentesis.

** Key words:**Temporomandibular joint, arthrocentesis, minimally invasive surgery.

## Introduction

Acute temporomandibular joint (TMJ) closed lock has traditionally been considered a consequence of the anterior or anteromedial displacement of an articular disc. The disc deforms, becomes impossible to reduce, and poses an obstacle to the normal movement of the mandibular condyle. Where conservative methods fail to resolve this clinical problem, surgery may be needed to restore mandibular function (1,2). However, since the TMJ arthroscopy concept gained popularity, Nitzan et al. (3) have drawn attention to the fact that the displaced disc in itself may not be entirely responsible for acute TMJ closed lock. This idea is based on the excellent results of arthroscopic lysis and lavage (4). It was held that the success of these procedures was not accompanied by any kind of change in the position or morphology of the disc (5). Consequently, it was speculated and then demonstrated to think that simply washing the upper articular space accounted for the success of arthroscopic surgery, rather than repositioning the disc (6). This cast a doubt on the idea of a displaced disc blocking condylar translation in all cases of acute lock.

Studies began on a phenomenon that can occur in joints at any stage of internal TMJ derangement. This phenomenon was characterised by a sudden, brusque, severe and persistent reduction in the maximum jaw opening to less than 25 mm, which was continuous but reversible by simple wash of the upper compartment. It occurs mainly when the disc conserves its normal biconcave shape and position and known as anchored disc phenomenon or anchored disc syndrome. The non-reducible disc could allow movement, in part, but could not account for this brusque phenomenon. It was thought that the restricted movement of the mandibular condyle on the articular eminence could be due to the reversible adhesion of a disc with normal morphology to the glenoid fossa, resulting from a suction cup phenomenon. It could produce by changes in the characteristics of synovial fluid. Later, this hypothesis was supported by the observation that in the upper articular compartment of blocked joints, there was greater negative intra-articular pressure than in unblocked joints (7,8). There are other historical events explaining the success of arthrocentesis. Many patients undergoing a TMJ arthrography (injection of a contrast medium to the upper and lower compartment) noticed less pain and an improvement in jaw movement following this procedure (9). It was also assumed that an important part of the effectiveness of surgical arthroscopy in treating acute severe lock could be due to the washing and elimination of chemical inflammation mediators rather than to the surgical instruments (10) applied directly to different arthroscopic findings, such as in perforation or synovitis (3,7).

## Material and Methods

A search was made on Medline in which the terms TMJ Arthrocentesis or TMJ minimally invasive surgery appeared. The result yielded 60 articles, 9 of which dealt with technical modifications, 16 with clinical research studies or synovial fluid analyses, and 3 of which were review articles. We selected 20 of the 32 remaining articles that complied with the scientific criteria proposed by us for the study design (number of patients, specific preoperative diagnoses, surgical techniques, monitoring, etc).

## Results

Several publications were found on arthrocentesis (6,10-28). The study design was variable ( Table 1): nine were prospective series, two were comparative prospective series, one was a long-term follow-up study, two were retrospective series, three were non-randomised studies, one was a double blind non-randomised study, one was a randomised, non double-blind study and another was a trial with randomisation. There was considerable heterogeneity with respect to the description of the symptoms treated: different Wilkes classifications of the dysfunction syndrome, anchored disc phenomena, disc positions with and without reduction, capsulitis or synovitis, acute and chronic cases, and osteoarthritis. In most cases, the inclusion criteria were explicit but the reporting of the exclusion criteria was not.

**Table 1:**
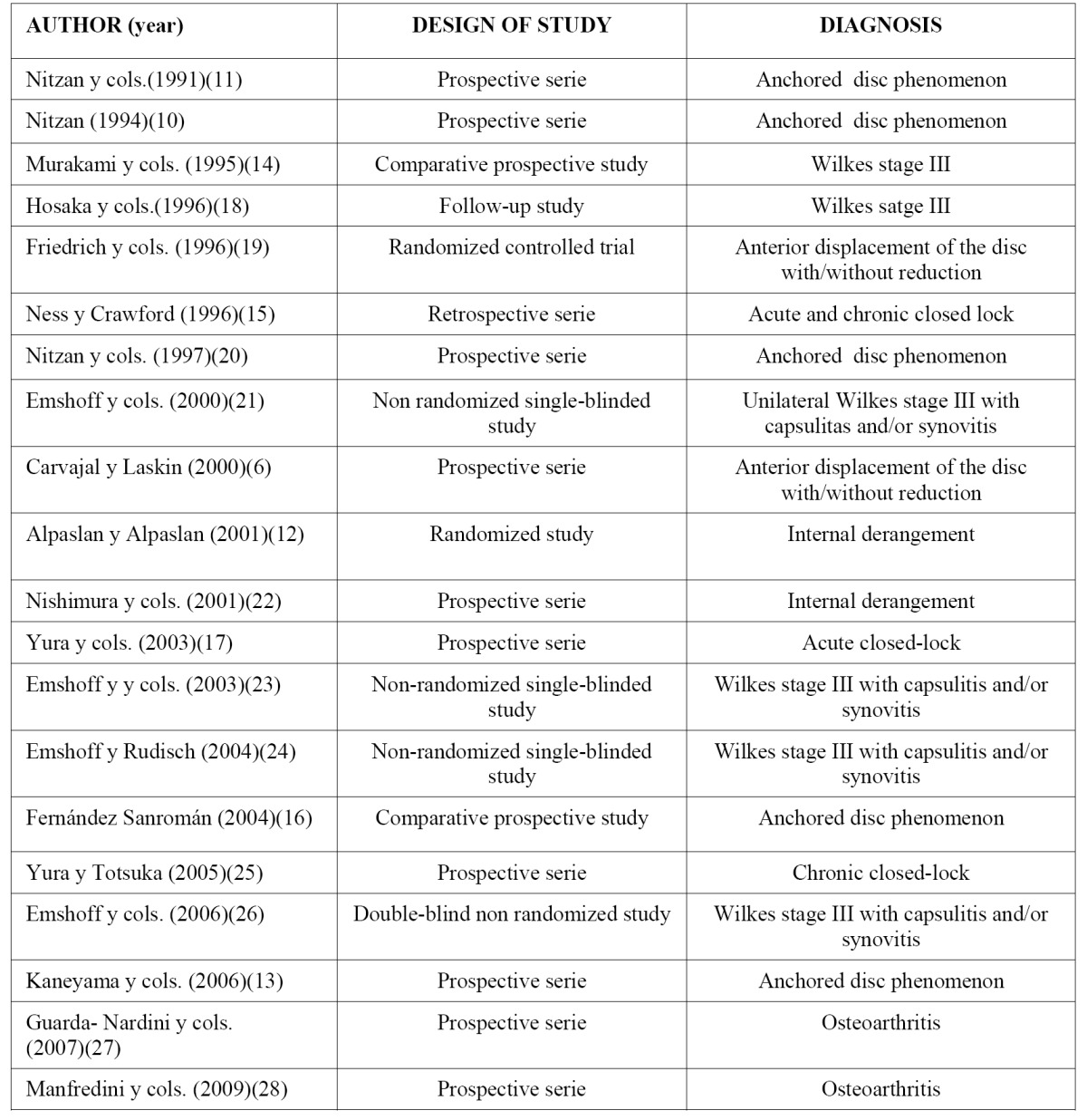
Design od the studies of the authors with diagnostic categories of the analyzed papers.

The patients were treated under local anaesthesia in 13 studies, two were treated with intravenous sedation, and general anaesthe-sia was employed in 5 studies ( Table 2). The volume of TMJ washing solution used was between 50 and 500 cc. In two studies (27,28), one arthrocentesis was performed every week, up to a total of five. The volumes used were 50 cc of serum and 1 cc of sodium hyaluronate. In 18 studies, the upper articular space was washed at low pressure, using a bag of elevated serum or a syringe. A compressor was used to increase the pressure of fluid entry into the joint in only two studies. After washing, corticosteroids were injected into the joint in 14 studies and sodium hyaluronate in 4 studies. No intra-articular medication was used in 2 studies. In practically all the studies, post-operative medication was administered, and an occlusal splint was recommended, with or without physiotherapy.

**Table 2:**
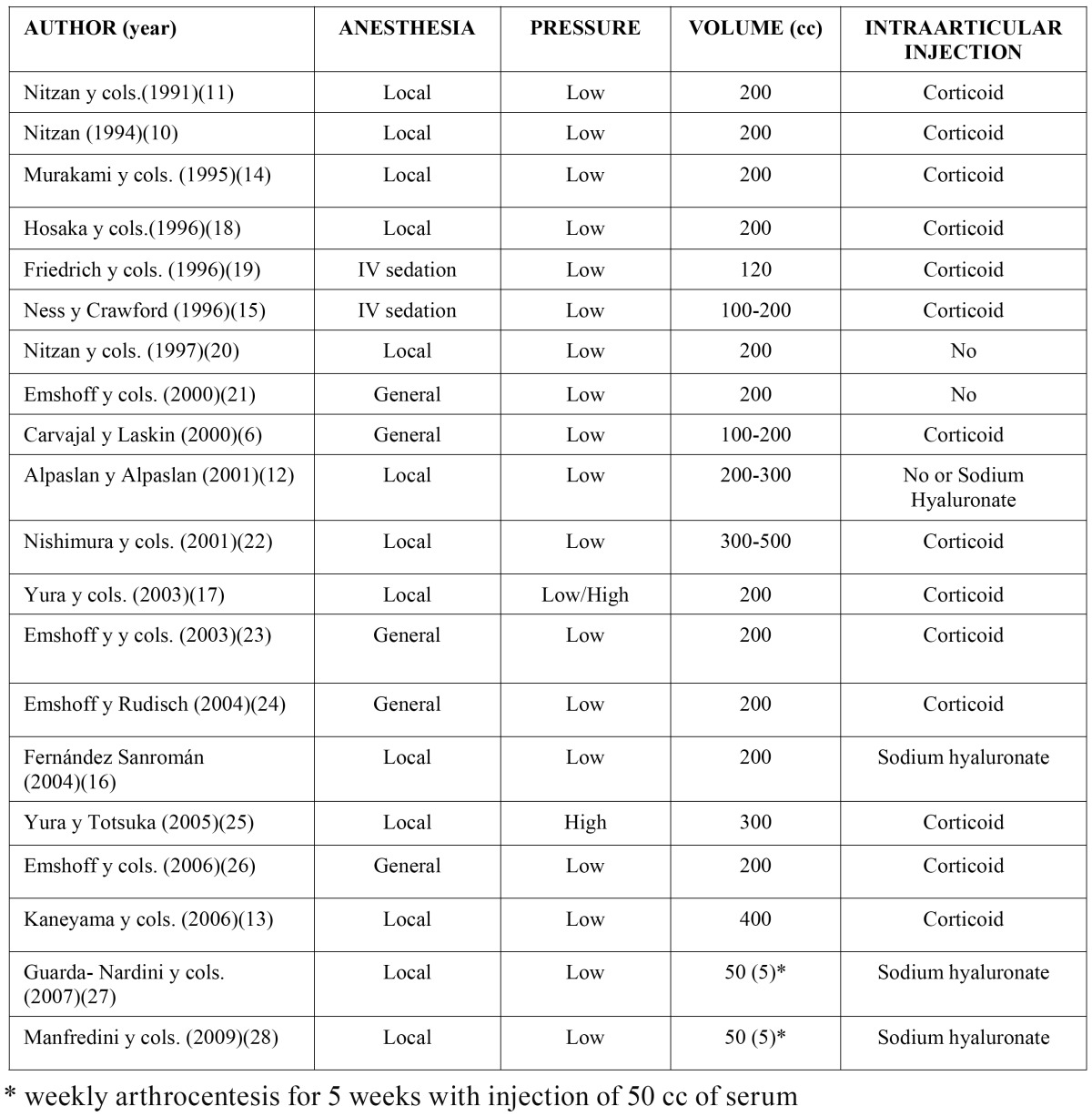
Treatment protocols: anestesia, pressure and volume of the liquid and intraarticular injection of substances.

Clinical follow-up of the patients in the studies was as follow: 3 years in one study (18), and a standard, non-prolonged follow-up procedure in 10 studies (6,10,11,13,15,16,20,22,26-28), one case lasting 6 months (14), five cases lasting 2 months, (22,25-29), one lasting one month (17) and a follow-up period of 24-36 months in two studies (12,19). In most studies, the criteria indicating successful results consisted of an improvement in maximum jaw opening and a reduction in pain level and mandibular dysfunction on an visual analogue scale (VAS). Additional criteria indicating success included the disappearance of joint pains and the normal diet of the patient in 3 studies (13,15,30).

After counting the number of articles reviewed on 612 joints in 586 patients, positive results were observed in 83.5%. The average age of the patients was 34.3 years and the average duration of the lock, 14 months ( Table 3). The average follow-up time was 12.8 months.

**Table 3:**
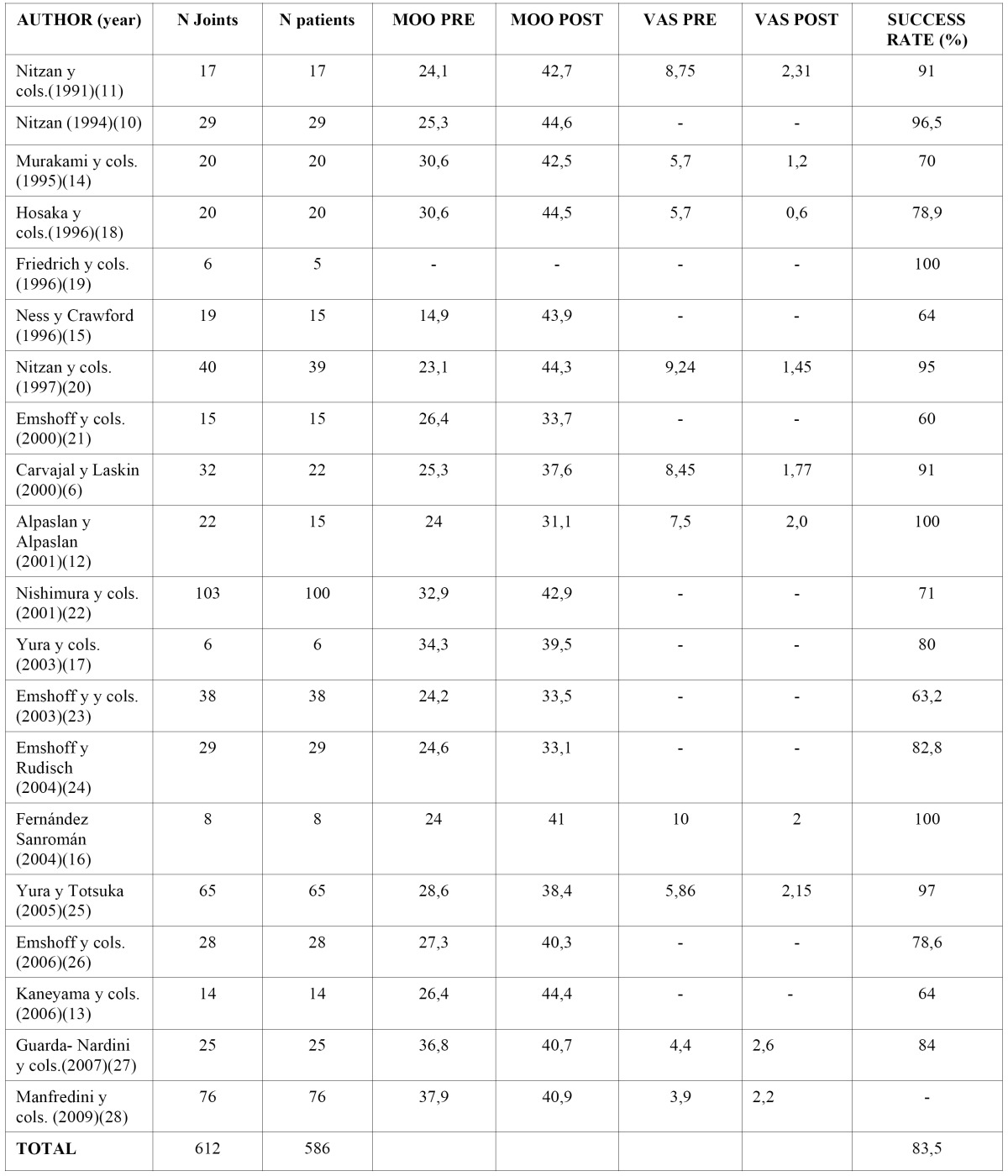
Summary of clinical results. MOO: Maximum oral opening. VAS: Visual analogue scale.

## Discussion

The results of the studies reviewed show that 612 joints with acute closed lock (disc displacement without reduction or anchored disc phenomenon) in 586 patients were successfully treated with arthrocentesis. The procedure can be executed safely under local anaesthesia, with or without intravenous sedation. Nevertheless, in 5 studies, the patients were treated under general anaesthesia. It is not clear whether this was a preference of the surgeon or of the patients. The overall success rate was 83.5%. Consequently, it appears reasonable to conclude that arthrocentesis is a simple, non-invasive, inexpensive and highly effective procedure, apart from having a low morbidity rate (10,12-16). Dimitroulis et al. (29) also suggest that arthrocentesis should be considered as an alternative to other, more invasive TMJ surgical procedures, provided it is applied to selected groups of patients.

Despite the finding that arthrocentesis was as effective as arthroscopic lysis and lavage in treating acute TMJ closed lock, Murakami et al. (14) conclude that arthrocentesis should not be regarded as an alternative to arthroscopic surgery. In this sense, Frost et al. (30) consider that Murakami’s stance is not reasonable, since they conclude that “the clinical efficacy of arthrocentesis may be somewhat less than that of arthroscopic surgery”. They consider arthrocentesis to be a therapeutic mode somewhere between non-surgical treatment and arthroscopic surgery. Hosaka et al. (18) maintain that if the therapeutic mechanism is not sufficiently clear and explained, clinical practitioners cannot be certain as to whether it can be considered an alternative procedure to surgery. However it is wrong to judge this the arthrocentesis as an alternative since it should always be used before any other procedure. Goudot et al. (31) also say that arthrocentesis and arthroscopy are equally effective methods in terms of pain, whereas arthroscopy is superior in terms of functional or mechanical results. On the other hand, Fernández Sanromán (16) says that arthrocentesis could be better indicated for treating patients who have been confirmed to be suffering anchored disc syndrome, and that arthroscopy could be the alternative, since it permits direct viewing of the pathological tissues and makes it possible to eliminate adherences.

The average age of the patients in 14 studies was 34.3 years, and the average duration of the lock was 12.8 months in 12 studies. In this respect, Murakami et al. (14) recommend indicating arthrocentesis in cases of acute TMJ closed lock not responding to non-surgical treatment. Patients approved for this treatment would be young patients suffering from this type of lock. It was concluded that age and the duration of the lock could be considered as indicators for predicting the result of this type of procedure. Nishimura et al. (22) also suggest that severe preoperative articular pain, relapse in the amount of the oral opening 1 week after arthrocentesis and preoperative condylar deformity may be negative factors in predicting the effectiveness of arthrocentesis. Nitzan et al. (20) say that the duration of the symptoms seems to affect the articular function, and although age does not affect the results of arthrocentesis, the pain and dysfunction values observed in patients aged over 40 indicate slower recovery. Emshoff and Rudisch (24) conclude that arthrocentesis in patients with chronic TMJ pain yields worse results in terms of pain reduction than in non-chronic patients. It´s also clear these patients suffer muscular pain that certainly will not respond to arthrocentesis.

In the studies reviewed, it is explained that patients suffering from acute TMJ closed lock were subjected to conservative treatment, to which they failed to respond and which they resisted for a reasonable length of time. This requisite is in accordance with the general indications of surgery on the TMJ. These indications signify that the indication of applying an invasive procedure, no matter how minimal, would not give the TMJ the opportunity to be repaired and to adapt (30). The act of applying an invasive procedure without any non-surgical therapeutic trial also eliminates the possible therapeutic success of that procedure. Fernández Sanromán (16) concludes that non-surgical treatment is not the correct option for treating anchored disc syndrome cases. Nitzan et al. (18) also suggest that arthrocentesis should be performed without delay in those patients in whom attempts to release the acute closed lock using non-surgical methods have failed. Likewise, they maintain that physiotherapy aimed at increasing the maximum jaw opening before arthrocentesis is not recommended in these patients, since, under pathological conditions, it may bring about the displacement of the condyle beneath the disc and cause structural damage to the joint.

Although adhesion to the upper articular space is one of the factors causing restrictions in the maximum jaw opening (17), patients with evidence of this were excluded from one study (11). It has been said that arthrocentesis may not always be recommendable for patients with severe adhesions. On the another hand, patients who are resistant to arthrocentesis may have fibrous adhesions in the TMJ (13). Yura et al. (25) studied this problem and found that low-pressure arthrocentesis was not successful in patients with severe anchorage, whereas high-pressure arthrocentesis was effective in breaking or releasing it. They concluded that irrigation at high pressure can eliminate adherences and increase articular space. Arthrocentesis may be a very effective procedure in patients with persistent or chronic closed lock and anchorage in the upper articular space. From another perspective, these authors say that the presence of anchorage in the upper articular space has no effect at all on the efficacy of high-pressure arthrocentesis and conclude that the effectiveness of this procedure is greater than that of low-pressure arthrocentesis. Nonetheless, the superiority of one technique over the other cannot be demonstrated, in particular owing to the absence of randomisation in both these studies. Furthermore, the success of low-pressure arthrocentesis is confirmed in the treatment of patients with acute TMJ closed lock, particularly patients with the anchored disc syndrome, where synovitis and the anchoring of the joint are the most common pathological symptoms observed (16).

No intra-articular injection was used in two studies. Corticosteroids were used in 14 studies and sodium hyaluronate in 4 studies, but in two of them, weekly arthrocentesis were applied, accompanied by sodium hyaluronate. Alpaslan et al. (12) found that patients with internal TMJ damage benefited from arthrocentesis with or without hyaluronate injections in terms of improving pain. However, they confirm that the results of arthrocentesis with hyaluronate appear to be better than arthrocentesis without intra-articular hyaluronate injections, particularly in patients suffering from acute TMJ closed lock. This was attributed to the faster and longer-lasting effect of hyaluronate in improving pain in these particular cases. The intra-articular hyaluronate injection seems to reduce the levels of nitric oxide and the substance reacting to the thiobarbituric acid in the synovial fluid. In contrast, there was no significant fall of these levels in patients receiving only the arthrocentesis(31). Both substances cause vasodilatation and vascular permeability, which may be associated with TMJ pain (32). The use of hyaluronate is currently a matter of controversy due to the short life of this product in the articular space (33). In other joints of the body, the ingestion of hyaluronate has not proven to exercise a marked therapeutic benefit compared to other conventional forms of treatment, and the high cost of the treatment may restrict its use. One bibliographic review concludes that, in view of the data obtained, the use of hyaluronate in arthrocentesis does not appear to yield conclusive information supporting the efficacy of this substance as compared to the absence of its use. From the scientific standpoint, better studies are called for in order to provide conclusive data (34).

The natural course of the internal TMJ damage showed that most patients are able to adapt to the abnormal disc position, and in this way, are able to maintain a relatively normal function (30,35). Hosaka et al.(16) report two cases classified as failures after 6 months of monitoring, in which the clinical signs and symptoms gradually improved after three years of monitoring. They suggest that this improvement could be the result of the natural course of the internal TMJ damage and not the initial therapeutic mode applied, arthrocentesis. Nishimura et al. (22) report that the success rate in their study could have been higher if the patients treated unsuccessfully with arthrocentesis, who underwent arthroscopic surgery 70 days after the arthrocentesis, had been evaluated at least 6 months after the arthrocentesis.

The specific therapeutic efficacy and scientific bases of arthrocentesis have not yet been validated. Several therapeutic concepts have been proposed to explain why washing the pathological fluid in the joint, followed by the elimination of the “suction cup effect” associated with adhesive force in locked joints, was also effective in non-locked joints (10,20,24,25). Unfortunately, there is little evidence to support the different proposals based on clinical or laboratory studies. These concepts could increase the degree of certainty in the therapeutic decision-making process (36).

Within the context of TMJ diseases, one logical parameter of success was considered to be “changing the impaired mandibular function in sufficient measure” as the result of restored movement and reducing pain in the TMJ. In most of the publications reviewed, improvement in maximum jaw opening and the reduction of pain levels and articular dysfunction on the VAS were the criteria used to define a successful result. However, these criteria were defined with considerable variability, and the precision limits of the measurement procedure applied were not described in any study. Many authors in the TMJ field use “success rate” to calculate the therapeutic result of their interventions, probably due to the lack of randomised clinical studies (37). These are usually based on conviction rather than on a research design proper and statistical analyses. Additional research is needed to respond to the question of how the results of TMJ arthrocentesis must be defined and documented, and how they must be used in disease-specific terms to quantify the efficacy of this procedure (38). Most of these publications can be criticised for their faulty methodology: non-randomising of patients, absence of control subjects, the partiality of their authors and inadequate monitoring. It could be considered that these same criticisms, applied to all studies on TMJ surgery, which require a high standard of randomised double blind trials, and clinical studies with placebo controls, are extremely difficult to circumvent (39). It is merely common sense to accept that randomised studies are the standard design for assessing therapeutic results.
